# National study of medical education, ethical attitudes and curricular exposure to voluntary assisted dying by VOICE (Views Of Incoming Clinicians on End-of-life care)

**DOI:** 10.1016/j.fhj.2026.100542

**Published:** 2026-06-03

**Authors:** Hamaad Ahmad Khan, Karanjot Chhatwal, Michael Bryan, Dev Gakhar, Rasi Mizori, Shreeya Mehta, Jawad Aziz, Harnoor Garha, Alisha Mahmud, Yusuf Alghabra, Rhieya Rahul, Jackson T.S. Cheung, Rami Sati, Issam Motairek, Fadi Adel, Stuart D. Rosen, Simon C. Cork, Sammy Arab, MedNex Research Collaborative

**Affiliations:** a School of Medicine, Anglia Ruskin University, Chelmsford, United Kingdom; b Imperial College School of Medicine, Imperial College London, South Kensington Campus, London, United Kingdom; c National Heart and Lung Institute, Imperial College London, Du Cane Road, London W12 0HS, United Kingdom; d Nuffield Department of Medicine, University of Oxford, Oxford, United Kingdom; e Leicester Medical School, College of Life Sciences, University of Leicester, Leicester, United Kingdom; f Faculty of Life Sciences & Medicine, King's College London, Guy's Campus, SE1 1UL London, United Kingdom; g School of Medicine, Royal College of Surgeons in Ireland (RCSI), Dublin, Ireland; h Faculty of Medical Sciences, University College London, Gower St, London WC1E 6BT, United Kingdom; i Department of Cardiovascular Medicine, Cleveland Clinic, Cleveland, OH, USA; j Department of Cardiovascular Medicine, Mayo Clinic, Rochester, MN, USA; k London Northwest University Healthcare NHS Trust, Uxbridge Road, Ealing, London W5 4NN, United Kingdom; l Northwick Park Hospital, Watford Road, Harrow, Middlesex HA1 3UJ, United Kingdom; m Department of Cardiovascular Medicine, Mayo Clinic, Rochester, MN, USA

**Keywords:** Assisted dying, Medical education, Ethics, End-of-life care, Medical students

## Abstract

**Background:**

Legislative moves to legalise assisted dying in the UK have renewed discussion on future clinicians’ preparedness for new potential responsibilities. This study examined medical students’ ethical attitudes, legal understanding and curricular exposure.

**Methods:**

VOICE was a national cross-sectional survey of UK and Ireland medical students in 2025 (n = 896). The questionnaire assessed ethical views, confidence, legal knowledge and teaching exposure. Descriptive statistics, thematic analysis and multivariable logistic regression explored predictors of ethical agreement, curriculum coverage and legal knowledge.

**Results:**

Most students (64.5%) believed that assisted dying can be ethically justified, yet legal knowledge was limited; only 7.6% correctly identified all eligibility criteria. Nearly 70% reported minimal curricular coverage. Formal teaching was associated with higher confidence and greater ethical agreement. Regional variation and concerns about coercion and inequalities were common.

**Conclusion:**

Students show broad ethical support but have very limited confidence, legal understanding and curricular preparation, highlighting the need for structured education.

## Highlights

• 64.5% of medical students see assisted dying as ethically justifiable.
• Legal knowledge is poor; 7.6% identified all eligibility criteria.
• 69.1% reported little or no curriculum coverage on assisted dying.
• Formal teaching boosts confidence to discuss assisted dying with patients.
• Students flagged risks of coercion and unequal access for vulnerable groups.

## Graphical abstract

**Figure:**
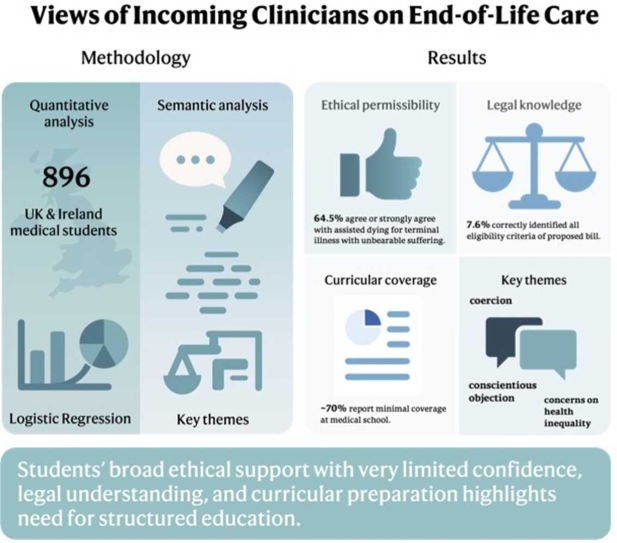

## Introduction

Assisted dying remains one of the most debated issues in medicine and raises practical challenges around capacity assessment, prognostication, documentation, and alignment with palliative care. At the time of writing, the House of Commons in the UK has passed the third reading of the Terminally Ill Adults (End of Life) Bill, proposing assisted dying for mentally competent adults with a prognosis of 6 months or less.^1^ This process would involve assessment by two independent doctors and a review panel’s oversight. Parallel proposals are under consideration in Scotland and Jersey.^2^, ^3^ These policy changes have major implications for clinical responsibilities.^4^

If enacted, today’s students would be among the first doctors practising under this framework, highlighting the need for coordinated curriculum development across medical schools and regulatory bodies. Existing literature offers limited insight into how prepared UK medical students are for these potential changes, with most studies focusing on qualified clinicians. Evidence is sparse on students’ understanding of current legal provisions, eligibility criteria and safeguarding concerns, or how they perceive risks to vulnerable groups.^5^, ^6^, ^7^ One Canadian survey suggests that medical students may be more supportive of assisted dying than practising doctors.^8^ Another Scottish study explored the perspectives of Scottish medical students on the proposed ‘Assisted Dying for Terminal Ill Adults (Scotland) bill’.^9^ Although this study demonstrated good breadth of support, it did not address students’ preparedness or education. Furthermore, there is limited research exploring UK medical students’ understanding of the current legal framework or the eligibility criteria proposed in recent UK bills. Evidence is also limited on whether students recognise risks to marginalised groups and widening health inequalities.^4^ These issues carry important safeguarding and ethical implications within assisted dying policy, and are thus highly important to address.

With no national guidance on teaching assisted dying, students may graduate holding strong ethical views but limited knowledge of legal and clinical requirements. This study examines students’ attitudes toward proposed legislative changes, their confidence discussing assisted dying, their understanding of the law, and the extent of curricular coverage. To our knowledge, it represents the first large-scale analysis of how ethical beliefs, legal understanding and educational exposure intersect during an active period of legislative debate (Supplementary Figure 1)**.**

## Material and methods

### Study design and setting

This was a UK-wide cross-sectional survey of medical students conducted while the Terminally Ill Adults Bill was progressing through early parliamentary stages (Supplementary Figure 2). The survey ran for 6 weeks (30 May – 12 July 2025) using a secure online platform (JISC Online Surveys). Participation was voluntary, anonymous and unincentivised, with all data stored in line with data protection standards.

### Participants and recruitment

Eligible participants were students enrolled in a primary medical qualification in the UK or Ireland, including UK citizens trained abroad. Irish students were included to allow comparison across neighbouring jurisdictions. Recruitment used a decentralised student-led approach via representatives, societies and national bodies. Only one response per participant was permitted, and no identifiable data were collected.

### Sample size

The achieved sample of 896 (Table 1) exceeded the planned minimum and provided sufficient power to explore associations within the volunteer cohort.

**Table 1:** Distribution of VOICE respondents by year and region of study (N = 896).

Group and category	n	%
**Year of study**		
Year 1	219	24.4
Year 2	179	20.0
Year 3	189	21.1
Year 4	133	14.8
Year 5	87	9.7
Year 6 (incl. intercalated)	89	9.9
**Region of study**		
East of England	36	4.0
London	267	29.8
Midlands	176	19.6
North East and Yorkshire	17	1.9
North West	78	8.7
Northern Ireland	49	5.5
Other (Please specify)	141	15.7
Republic of Ireland	22	2.5
Scotland	28	3.1
South East	52	5.8
South West	15	1.7
Wales	15	1.7

### Survey development

Items were informed by literature on assisted dying, policy statements and national surveys. The final 28-item questionnaire took approximately 5–7 min to complete (Supplementary Table 1). A brief introductory section outlined definitions and legislative context to ensure consistent interpretation. One open-ended item captured additional reflections.

### Data processing

Responses were exported in .csv format. Incomplete entries, those without consent and timestamp-linked duplicates were removed. Free-text entries were screened for identifiable information. Multiple-response items were binarised, Likert items numerically coded, and missing data excluded pairwise.

### Statistical analysis

Descriptive statistics summarised demographic characteristics and key outcomes. Five hypotheses were specified *a priori*, before data lock and before any regression model was fitted. H1 (curricular exposure → coverage): formal medical-school teaching on assisted dying is associated with lower odds of reporting minimal curriculum coverage, adjusted for year of study, region of training, source of learning and self-reported awareness/understanding (Supplementary Table 2). H2 (ethical attitudes): agreement that assisted dying is ethically justifiable in cases of terminal illness with unbearable suffering is independently associated with formal curricular exposure, year of study and region of training, adjusted for self-reported confidence and legal-knowledge score (Supplementary Table 3). H3 (personal participation): intention to opt out on ethical or religious grounds is independently associated with region of training, year of study, source of learning, confidence and legal-knowledge score (Supplementary Table 4). H4 (factual knowledge): the three legal-knowledge items (Q25–Q27) differ in accuracy, tested by two-proportion z-tests. H5 (teaching → confidence): self-reported confidence in legal/ethical communication is higher among students who have received formal teaching, tested by a two-proportion z-test. Descriptive one-sample z-tests compared observed proportions against 50%, with 95% Wald confidence intervals. Group differences were assessed using Pearson’s chi-squared tests for categorical variables and Kruskal–Wallis tests for ordinal data. Survey items were recoded into binary or grouped categories using the pre-specified thresholds set out under Model specification below. Within each model family, p-values were adjusted for multiplicity using the Benjamini–Hochberg false-discovery-rate procedure; both raw and adjusted p-values are reported in the Supplementary Tables. Analyses were performed on complete cases only, with no imputation. Statistical significance was defined as p < 0.05.

Three multivariable binary logistic regression models tested H1–H3. All models were pre-specified; no stepwise selection was performed. Models were fitted by maximum likelihood using the statsmodels Logit implementation in Python 3.11. Adjusted odds ratios (aOR) were obtained as exp(β) and 95% confidence intervals as exp(β ± 1.96·SE). Outcomes were binarised as follows: minimal curriculum coverage (Supplementary Table 2, n = 886) was defined as Q11 ∈ {ʻNot at all’, ʻSlightly’} = 1 vs. {ʻModerately’, ʻWell’, ʻExtensively’} = 0; ethical agreement (Supplementary Table 3, n = 896) was Q16 ∈ {ʻAgree’, ʻStrongly agree’} = 1 vs. else = 0; ethical/religious opt-out (Supplementary Table 4, n = 883) was Q20 = ʻYes’ = 1 vs. {ʻNo’, ʻUnsure’} = 0. Covariates, identical across models except where noted, were: year of study (six levels; reference: Year 1), region of training (13 levels; reference: East of England), source of learning for assisted dying (Q8, a multi-response item entered as four independent binary indicators (academic curriculum, medical school teaching, media/news, personal experience) and a ʻnot learned about’ indicator, each coefficient representing the contrast of reporting-that-source vs. not-reporting-it), and self-reported awareness (Q6 UK Bill familiarity and Q7 eligibility understanding, in the curriculum-coverage model) or self-reported confidence and curriculum coverage (Q10 and Q11, in the ethical-agreement and opt-out models), each entered as a continuous 1–5 Likert score so that the aOR is interpretable per one-point increase. Reference categories were chosen before fitting and held constant across tables to allow cross-table comparison. Records missing any predictor or outcome were excluded by listwise deletion (ST2: 10 excluded; ST3: 0 excluded; ST4: 13 excluded). Within each model family, p-values were adjusted for multiplicity using the Benjamini–Hochberg procedure; raw and adjusted p-values are reported. Model fit was assessed by the likelihood-ratio test against the null model (reported at the foot of each Supplementary Table), McFadden pseudo-R^2^, and the Hosmer–Lemeshow test for calibration; variance inflation factors were inspected for all covariates and were <2.5 throughout, indicating no problematic collinearity. Full model specifications and code are available at https://github.com/MichaelEBryan/VOICE.

## Results

### Medical student exposure to assisted dying in education and training

**Fig. 1:**
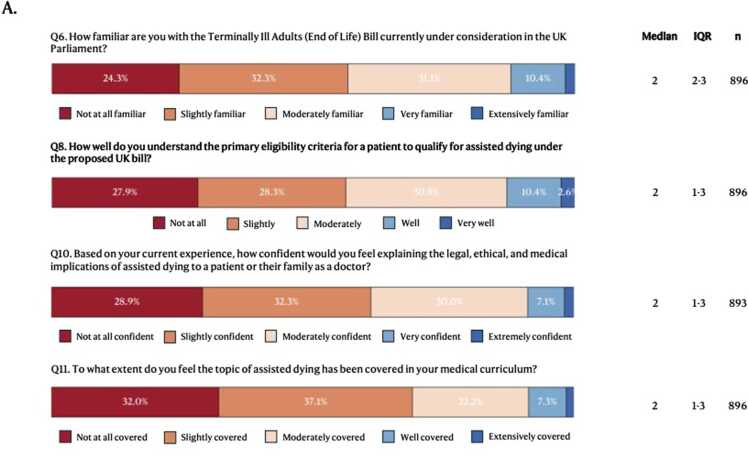
Medical student exposure to assisted dying in education and training. Likert-scale distributions of self-reported familiarity with the Terminally Ill Adults (End of Life) Bill (Q6), understanding of eligibility criteria (Q8), confidence in discussing the legal, ethical and medical implications of assisted dying with patients or families (Q10), and extent of curricular coverage (Q11), across the full sample (n = 893–896). Median and interquartile range (IQR) reported for each item.

We first examined students’ self-reported confidence in legal, ethical and medical conversations (Fig. 1). Across the national sample, confidence was low. Only 8.8% of students described themselves as very or extremely confident, while 61.1% reported either no confidence or only slight confidence in these discussions. When asked whether their curriculum had adequately covered assisted dying, 69.1% reported either no coverage (32.0%) or only slight coverage (37.1%). Just 1.5% considered the topic extensively covered. Confidence varied significantly by year of study (P < 0.001) but not by geographic region (P = 0.716) (Supplementary Figure 3A).

When examining where students acquire information, personal reading (27.0%), media coverage (25.8%) and conversations with peers or mentors (20.2%) were more prevalent sources of information than formal medical school teaching (21.2%). Notably, 4.0% of respondents reported having not learned about assisted dying at all (Supplementary Figure 3B).

Students who reported receiving any formal instruction on assisted dying during medical school were significantly more likely to express high confidence in discussing its legal, ethical and medical implications with patients or families (Supplementary Figure 3C). In multivariable logistic regression, formal teaching remained a significant independent predictor of high confidence (OR = 1.60; 95% CI: 1.08–2.39; p = 0.020, Supplementary Table 2), whereas informal learning sources, including media or personal experiences, were not.

### Medical student views on the ethical justifiability and scope of assisted dying

**Fig. 2:**
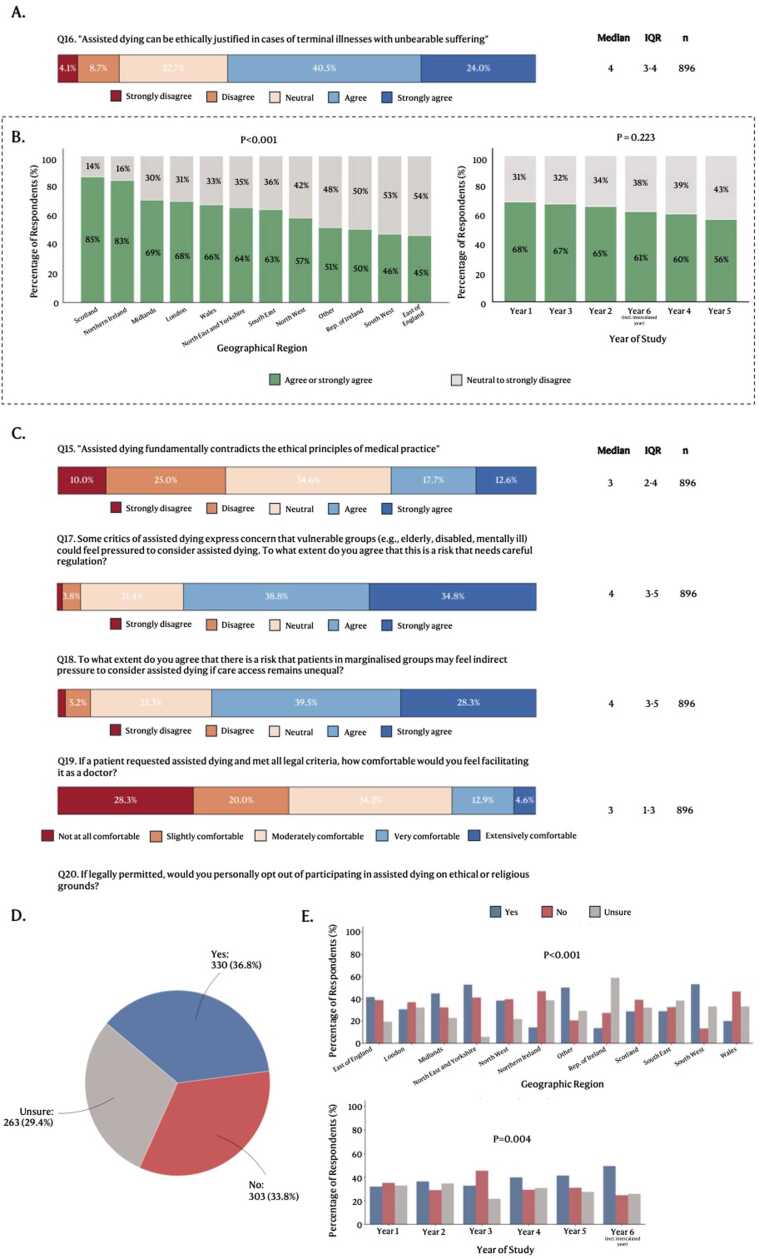
Medical student views on the ethical justifiability and scope of assisted dying. (A) Likert-scale distribution of agreement with the ethical justifiability of assisted dying in terminal illness with unbearable suffering (Q16; n = 896). (B) Proportion agreeing or strongly agreeing with ethical justifiability by geographical region (left; p < 0.001) and year of study (right; p = 0.223). (C) Likert-scale distributions of views on ethical contradiction with medical practice (Q15), risks to vulnerable groups (Q17), risks of indirect pressure in marginalised groups (Q18) and comfort facilitating assisted dying (Q19). (D) Pie chart showing proportions of students who would personally opt out of participating in assisted dying on ethical or religious grounds (Q20; n = 896). (E) Opt-out intent by geographical region and year of study (p < 0.001 and p = 0.004, respectively).

A majority of UK medical students support the ethical permissibility of assisted dying in certain contexts. When asked whether assisted dying could be ethically justified in cases of terminal illness accompanied by unbearable suffering, 64.5% agreed or strongly agreed (Fig. 2A).

Ethical attitudes varied by region (Fig. 2B). In adjusted models, students based in Scotland and Northern Ireland had substantially higher odds of agreeing with ethical justification than those in the East of England (reference region; Supplementary Table 3), with aOR estimates of 7.10 (95% CI: 1.99–25.28; p = 0.002) for Scotland and 6.30 (95% CI: 2.18–18.17; p < 0.001) for Northern Ireland. London (aOR = 2.61; 95% CI: 1.26–5.47; p = 0.010) and the Midlands (aOR = 2.86; 95% CI: 1.34–6.11; p = 0.007) also showed elevated support. The wide confidence intervals for Scotland and Northern Ireland reflect modest regional sub-sample sizes and should be interpreted as indicating that support is somewhere between two- and 20-fold higher rather than as precise point estimates; the direction of effect, however, is consistent across models. This regional pattern persisted after adjusting for year of study, exposure to teaching and legal knowledge, consistent with sociocultural and institutional variation in the moral climate of UK medical education.

Comfort with facilitating assisted dying was more divided (Fig. 2C–D), with 17.8% describing themselves as very or extremely comfortable, while 48.3% were not at all or only slightly comfortable. Significantly, 36.8% of students indicated that they would opt out of participating in assisted dying on ethical or religious grounds, even if it became legally permitted (Fig. 2D).

No monotonic pattern emerged across years of study. Students in Year 5 had lower odds of agreeing with ethical justification than Year 1 peers (aOR = 0.52; 95% CI: 0.30–0.90; p = 0.022; Benjamini–Hochberg-adjusted p = 0.121), but Year 4 and Year 6 did not differ significantly, and point estimates were not ordered by year. We therefore avoid a developmental interpretation and note only that Year 5 emerged as an outlier; potential explanations include the distinctive clinical-placement profile of that year in many UK curricula, but cross-sectional data cannot distinguish such an effect from a cohort effect.

### Perspectives on training, safeguards, and ethical concerns

Nearly three-quarters of students (73.7%) agreed or strongly agreed that assisted dying poses risks of pressure on vulnerable groups, such as older or mentally ill people, that require careful regulation. A similar majority (67.9%; 608/896) expressed concern that unequal access to care could create indirect pressure on marginalised patients to consider assisted dying.

Intent to opt out varied by region (unadjusted p < 0.001), with the lowest rates observed in the Republic of Ireland and Northern Ireland (Fig. 2E). In adjusted models, students in Northern Ireland (aOR = 0.22; 95% CI: 0.07–0.67; raw p = 0.008; BH-adjusted p = 0.089) and the Republic of Ireland (aOR = 0.21; 95% CI: 0.05–0.84; raw p = 0.028; BH-adjusted p = 0.154) had lower odds of opting out compared to those in the East of England (Supplementary Table 4). The direction of these effects mirrors those observed for ethical agreement, although after multiplicity correction only the direction (not the significance) is robust for these two regions; the pattern is nevertheless consistent with local cultural and educational contexts shaping professional willingness to participate.

### Medical student knowledge of safeguards, exclusion criteria, and inclusion criteria

Despite high levels of ethical support, factual knowledge of assisted dying legislation was limited. On a three-item assessment, only 7.6% of respondents (49/645) answered all questions correctly regarding eligibility criteria, while 16.7% (105/628) fully answered questions on legal status, and 8.5% (53/627) correctly identified where assisted dying is legal (Fig. 4A). Denominators vary because some respondents skipped individual items. Even in later years of training, fewer than 10% of students achieved full accuracy on key legal criteria (Fig. 4B).

**Fig. 3:**
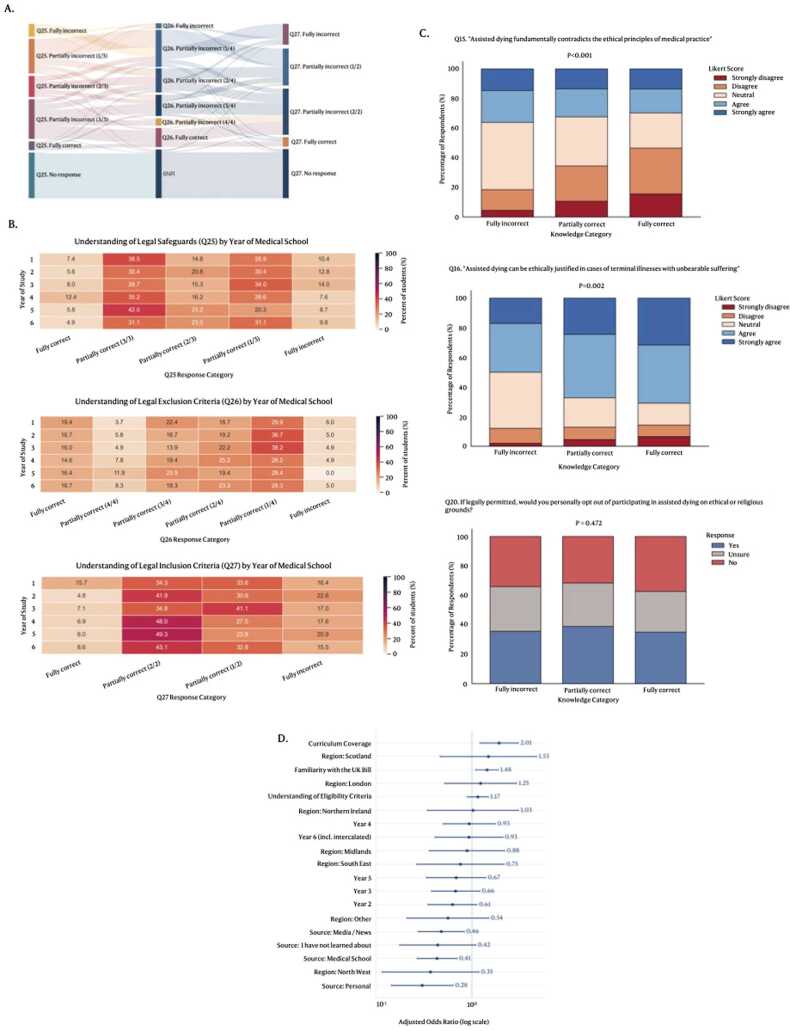
Medical student knowledge of safeguards, exclusion criteria, and inclusion criteria in the proposed UK Assisted Dying Bill. (A) Alluvial diagram illustrating consistency of responses across knowledge items on safeguards (Q25), exclusion criteria (Q26), and inclusion criteria (Q27). (B) Heatmaps showing distribution of response categories for Q25, Q26 and Q27 by year of study. (C) Stacked bar charts showing ethical attitudes stratified by legal knowledge category (fully incorrect, partially correct, fully correct) for Q15 (p < 0.001) and Q16 (p = 0.002) and opt-out intent by knowledge category (Q20; p = 0.472). (D) Forest plot of adjusted odds ratios from multivariable logistic regression identifying predictors of full accuracy on eligibility criteria (Q27).

Students with more accurate understanding of eligibility criteria were significantly more likely to agree that assisted dying is ethically justifiable in cases of terminal illness with suffering (p = 0.002), and less likely to view it as ethically contradictory (p < 0.001; (Fig. 3C). However, neither legal knowledge (OR = 1.01) nor self-reported ethics curriculum coverage (OR = 1.03) remained significant predictors in multivariable models of ethical support (Fig. 3D). Informal and personal sources of information, including media, peers and lived experience, were strongly associated with reduced support for legalisation (eg, personal source: OR = 0.28; media/news: OR = 0.46).

**Fig. 4:**
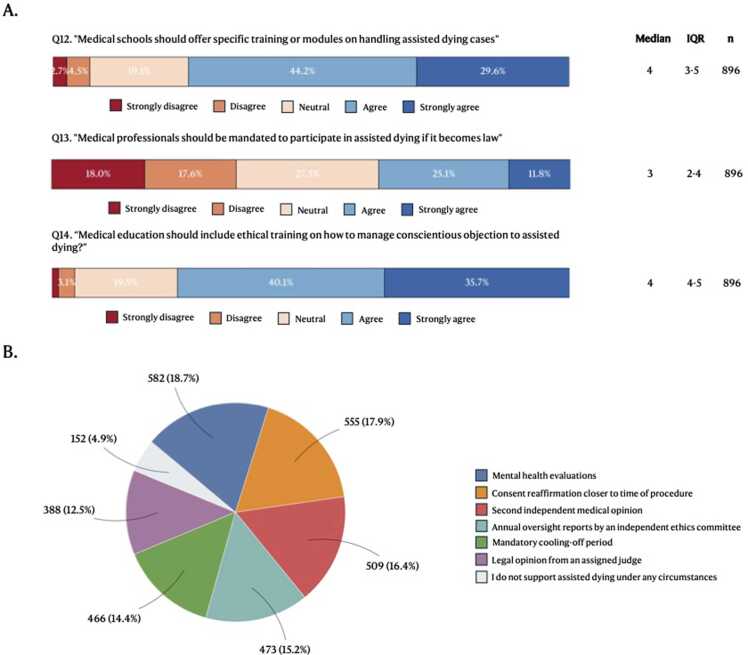
Perspectives on training, safeguards, and ethical concerns regarding assisted dying. (A) Likert-scale distributions of agreement with statements on medical school training (Q12; n = 896), mandatory participation (Q13; n = 896), and conscientious objection training (Q14; n = 896). (B) Pie chart showing the distribution of safeguards students would support if assisted dying were legalised in the UK (Q24; n = 896; multiple responses permitted).

There was broad consensus on the importance of preparing for conscientious objection. 75.8% of students (679/896) supported the inclusion of formal ethics training on how to manage conscientious objection to assisted dying (Fig. 4A). Mental health evaluations (18.7%), consent reaffirmation closer to the time of procedure (17.9%) and second independent medical opinions (16.4%) were the most commonly chosen safeguards (Fig. 4B).

### Thematic analysis of free-text responses (Supplementary Figure 2)

Open-text responses highlighted a widespread perception among medical students that current training on assisted dying is insufficient. Many described having ʻnever been taught this in medical school’ or learning only ʻfactual details’ without opportunities to explore ethical implications. Students consistently expressed a desire for earlier, more structured teaching. This gap left many feeling unprepared for possible future responsibilities.

Concerns about coercion and structural inequality were widespread. Respondents repeatedly linked these concerns to broader health system failures, such as the underfunding of palliative care. Others pointed to the potential for assisted dying to widen health inequalities, particularly where access to high-quality end-of-life care varies by socioeconomic status. Several highlighted the risk that patients with mental illness, disability or chronic pain might face disproportionate exposure to this option if not properly safeguarded.

Professional and legal fears featured prominently, with many students questioning whether regulatory bodies would protect clinicians involved in assisted dying. Others raised uncertainty about how assisted dying would intersect with the duty to ‘do no harm’ and whether participating in such care might expose them to future complaints or moral distress. Calls for mental health support for providers were common.

Students were divided on the ethics of assisted dying, but nearly all agreed that it should remain a matter of personal conscience. Many insisted that participation must never be compulsory. While some viewed assisted dying as a ʻmercy’ for those experiencing intractable suffering, others warned of a ʻdangerous precedent’ and feared that eligibility criteria would ʻgradually expand’.

## Discussion

This national study of medical students across the UK and Ireland suggests that the next generation of doctors are ethically open to assisted dying, but under-trained, under-informed and concerned about its practical implementation. Assisted dying is not yet codified into UK law and is not currently mandated within the General Medical Council’s Outcomes for Graduates; however, the legislation passing key parliamentary stages signals a possible imminent shift in clinical responsibilities. Undergraduate education has a critical role in proactively preparing students for the legal and ethical realities that they may encounter, but this must be achieved through considered prioritisation within a finite curriculum. Early, longitudinal exposure to these evolving debates ensures that future clinicians are not only legally literate, but also equipped to engage meaningfully with patients, colleagues and policy in a rapidly changing landscape. The VOICE study provides the first large-scale mapping of the ethical, educational and legal landscape shaping future doctors’ readiness to navigate assisted dying.

The findings of this study suggest that students are forming strong ethical positions in the absence of legal literacy or clinical training. For a generation likely to face legalised assisted dying during their careers, this risks eroding the safety and legality of end-of-life care.

Students who had received dedicated instruction on assisted dying were more likely to support its ethical justification. While media and lived experience influence students supporting assisted dying, they do not equip them with the skills to discuss it confidently. This suggests that the academic curriculum exerts a unique influence on ethical positioning. Over two-thirds of students felt that assisted dying was either not covered or only slightly covered in their training.

Geography emerged as a significant and under-recognised driver of ethical attitudes. Students in Scotland and Northern Ireland had markedly higher odds of supporting ethical justification for assisted dying than those in the East of England (aOR 6–7, 95% CI spanning roughly two- to 25-fold), even after adjusting for curricular exposure and legal knowledge. This is a large and directionally robust effect, albeit imprecisely estimated in these smaller regional strata, suggesting sociocultural or institutional differences in the ethical climate across UK and Ireland medical education.

Legal knowledge and ethics education did not independently predict students’ ethical views. Ethical positions appear to develop outside the boundaries of the curriculum and may not shift in response to knowledge alone. For medical educators, this presents a clear challenge: factual teaching must be complemented by opportunities for reflection, open discussion, and engagement with real-world ethical dilemmas.

Concerns about coercion and structural inequality were pervasive across the cohort, echoing critiques raised by disability rights advocates.^10^, ^11^ Rather than framing assisted dying solely as a matter of individual autonomy, students recognise the contextual pressures and structural inequalities that could distort patient choice.

Despite broad ethical support, students were divided on whether they would personally participate in assisted dying if it became legal. The data further suggest that support for legalisation does not automatically translate into professional willingness. Many students appear to draw a line between what they believe should be permitted and what they themselves would be comfortable enacting. This distinction, between policy support and personal participation, is under-theorised in current medical ethics education, and deserves greater attention.

Findings further report overwhelming support for training on conscientious objection. A coordinated national framework involving regulatory colleges, professional societies and palliative care organisations may help standardise education to support practising trainees and future clinicians.

The implications of these findings extend beyond curriculum design. Any proposal to expand undergraduate teaching on assisted dying must be considered within the context of an already densely packed medical curriculum. Introducing new content in this area will likely require prioritisation and potential de-emphasis of some existing topics, rather than simple curricular expansion. This raises a broader question for educators and regulators: how emerging, policy-relevant domains such as assisted dying should be balanced against established curricular components. Given the immediacy of potential legislative change and the clear gaps in legal understanding and preparedness identified in this study, there is a strong argument that such content represents a necessary evolution of the curriculum, rather than an optional addition. They touch on the professional identity of a new generation of doctors who will be asked to deliver care under legal regimes still in flux. If England and Wales legalises assisted dying, the burden of implementation will fall not on legislators but on clinicians. This study shows that UK medical education is not yet prepared for that challenge. But it also shows that students are asking for guidance in how to practice ethically, legally and equitably in a changing world.

The study’s primary strength is its breadth and depth. With nearly 900 respondents across all UK nations, all stages of medical training and a wide range of institutional settings, the VOICE study captures a panoramic view.

Equally important is the study’s multidimensional design. The inclusion of validated statistical methods, from hypothesis testing to multivariable logistic regression, provides rigour and the consistent use of confidence intervals allows for transparent reporting of uncertainty. One of the study’s most novel features is its use of legal knowledge. This identified a compelling disjunction between moral support for assisted dying and the ability to identify who is eligible, where the practice is legal, or what current UK law permits.

However, as with all cross-sectional surveys, causality cannot be inferred. The observed decline in support among later-year students could reflect greater clinical scepticism, but may also be confounded by cohort effects. These trends, while striking, require longitudinal follow-up to establish directional change over time.

Selection bias also remains a possibility. While our sample includes a broad spectrum of beliefs and demographics, and captures students from a wide range of medical schools and regions, it may still under-represent less engaged or less confident respondents. The absence of weighting by medical school size or stratification by institutional characteristics also limits generalisability to the full UK student population. That said, the diversity and balance observed across demographic and attitudinal variables offer some reassurance of external validity.

Our sample reflects a pragmatic trade-off: large, representative probability samples are ideal but often impractical, whereas accessible multi-institution volunteer samples remain open to self-selection bias. Accordingly, our findings should be interpreted as indicative of patterns rather than precise national prevalence estimates.

Some limitations arise from the nature of self-reporting. Confidence, willingness to participate and exposure to teaching are inherently subjective. They are likely to be influenced by context, memory and local educational norms. Legal understanding is fragile under pressure, and while our knowledge quiz improves on prior self-assessment tools, it does not replace formal legal education or clinical application. Furthermore, our legal knowledge questions were based on provisions from an evolving bill; consequently, students’ answers may reflect shifting proposals rather than persistent gaps.

Finally, this study does not attempt to map the full ethical terrain of assisted dying. It focuses on a narrow but policy-relevant subset, terminal illness with suffering, consistent with recent UK legislative proposals. Despite limitations, the VOICE study stands as a potentially beneficial contribution to UK medical ethics education research.

## Conclusion

This survey shows that although UK and Ireland medical students largely support the ethical permissibility of assisted dying, many lack confidence, legal understanding and adequate curricular preparation. Addressing these gaps will require structured teaching, opportunities for open discussion, and integration of evolving legislation into undergraduate training. If assisted dying becomes lawful, medical schools and national bodies will need to develop coordinated approaches to prepare future clinicians for its safe and ethical implementation.

## CRediT authorship contribution statement

**Issam Motairek:** Writing – review & editing, Validation, Supervision, Methodology. **Fadi Adel:** Writing – review & editing, Validation, Supervision, Methodology. **Dev Gakhar:** Writing – review & editing, Writing – original draft, Validation, Methodology. **Stuart D. Rosen:** Writing – review & editing, Validation, Supervision, Methodology. **Rasi Mizori:** Writing – review & editing, Investigation, Data curation. **Simon C. Cork:** Writing – review & editing, Validation, Supervision, Methodology. **Shreeya Mehta:** Writing – review & editing, Investigation, Data curation. **Sammy Arab:** Writing – review & editing, Visualization, Validation, Supervision, Methodology, Formal analysis, Conceptualization. **Jawad Aziz:** Writing – review & editing, Investigation, Data curation. **Harnoor Garha:** Writing – review & editing, Investigation, Data curation. **Alisha Mahmud:** Writing – review & editing, Investigation, Data curation. **Yusuf Alghabra:** Writing – review & editing, Investigation, Data curation. **Rhieya Rahul:** Writing – review & editing, Investigation, Data curation. **Jackson T.S. Cheung:** Writing – review & editing. **Hamaad Ahmad Khan:** Writing – review & editing, Writing – original draft, Methodology, Investigation, Data curation, Conceptualization. **Rami Sati:** Writing – review & editing. **Karanjot Chhatwal:** Writing – review & editing, Writing – original draft, Visualization, Methodology, Investigation, Data curation, Conceptualization. **Michael Bryan:** Writing – review & editing, Writing – original draft, Supervision, Software, Formal analysis.

## Ethics approval and consent to participate

Ethics approval was granted by Anglia Ruskin University’s Research Ethics Committee (Reference: ETH2425-6140). All procedures adhered to the Declaration of Helsinki. Participation was voluntary; all respondents received study information and provided electronic informed consent prior to accessing the survey. No minors were enrolled.

## Funding

This research did not receive any specific grant from funding agencies in the public, commercial or not-for-profit sectors.

## Declaration of competing interest

The authors declare the following financial interests/personal relationships which may be considered as potential competing interests: Professor Rosen is an associate editor of this journal. The authors declare no other competing financial interests or personal relationships that could have appeared to influence the work reported in this paper.

## Data Availability

Due to the sensitive nature of the topic and to protect participant anonymity, raw survey data are not publicly available. The dataset includes free-text responses and demographic combinations that may permit re-identification. De-identified, aggregated data may be made available upon reasonable request to the corresponding author, subject to approval by Anglia Ruskin University’s research governance procedures. Code for data processing and analysis is available at: https://github.com/MichaelEBryan/VOICE.

## Footnotes

Appendix A Supplementary data associated with this article can be found in the online version at doi:10.1016/j.fhj.2026.100542.
