# Supplementary material for: National study of medical education, ethical attitudes and curricular exposure to voluntary assisted dying by VOICE (Views Of Incoming Clinicians on End-of-life care)
Source: Future Healthc J. 2026 Jun 3;13(3):100542. doi: 10.1016/j.fhj.2026.100542 (PMC13315502; doi:10.1016/j.fhj.2026.100542)
Supplement: Supplementary file 1 — Supplementary material [file mmc1.docx]

**Supplementary Figure 1.** Overview of the VOICE Study

**VOICE**

(**V**iews **O**f **I**ncoming **C**linicians on assisted dying and **E**nd-of-life safeguards)


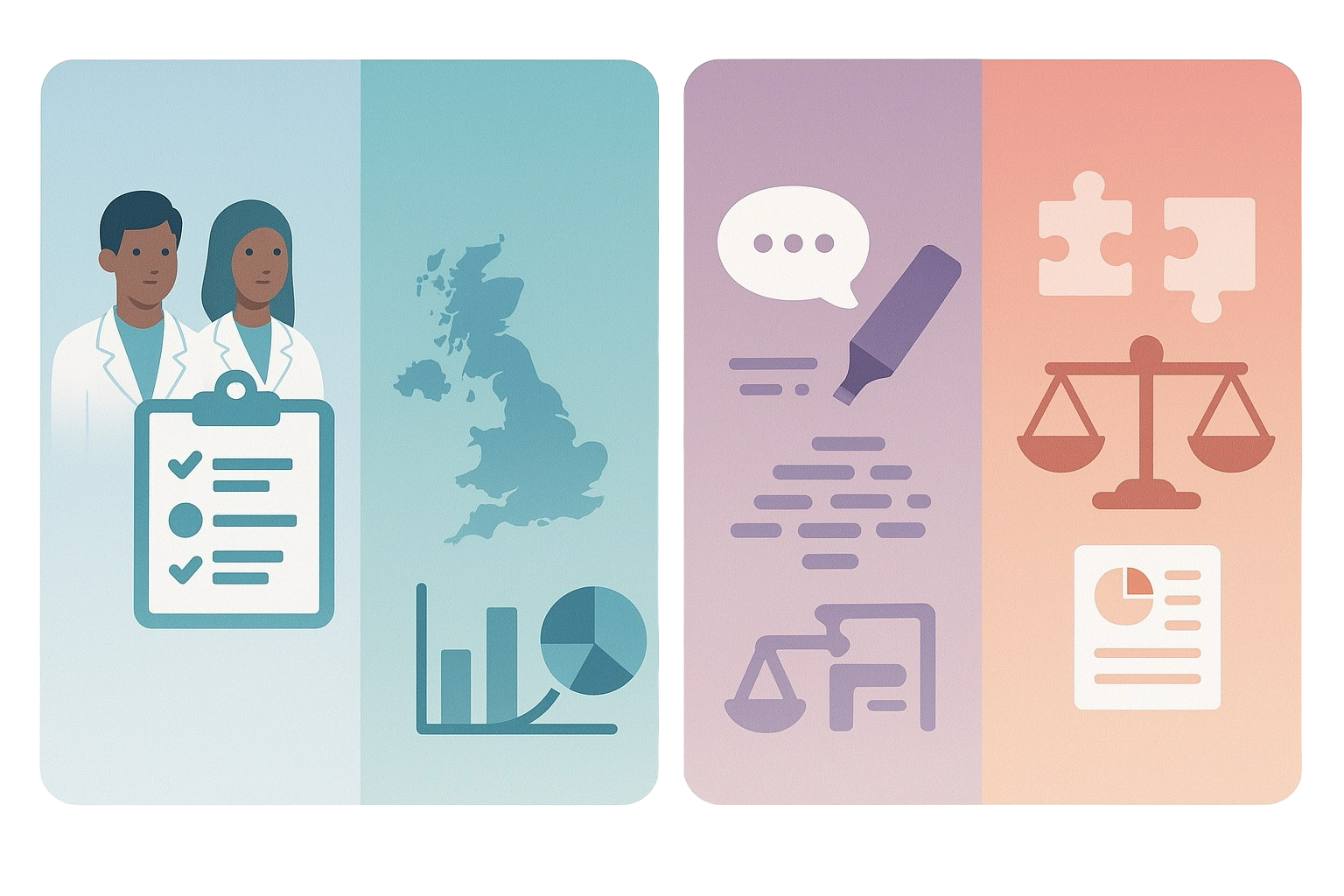
UK & International Medical Students

896 surveyed for attitudes towards

end-of-life safeguards

Quantitative Analysis

Logistic Regression

Semantic Analysis

Key Themes

Integration

Recommendations

**Supplementary Figure 1. Overview of the VOICE Study.**

Schematic summarising the study design, including participant population (UK and Ireland medical students, n=896), analytical approaches (quantitative analysis and logistic regression; semantic analysis and key themes), and outputs (recommendations).

**Supplementary Figure 2.** UK Representation of the VOICE Study


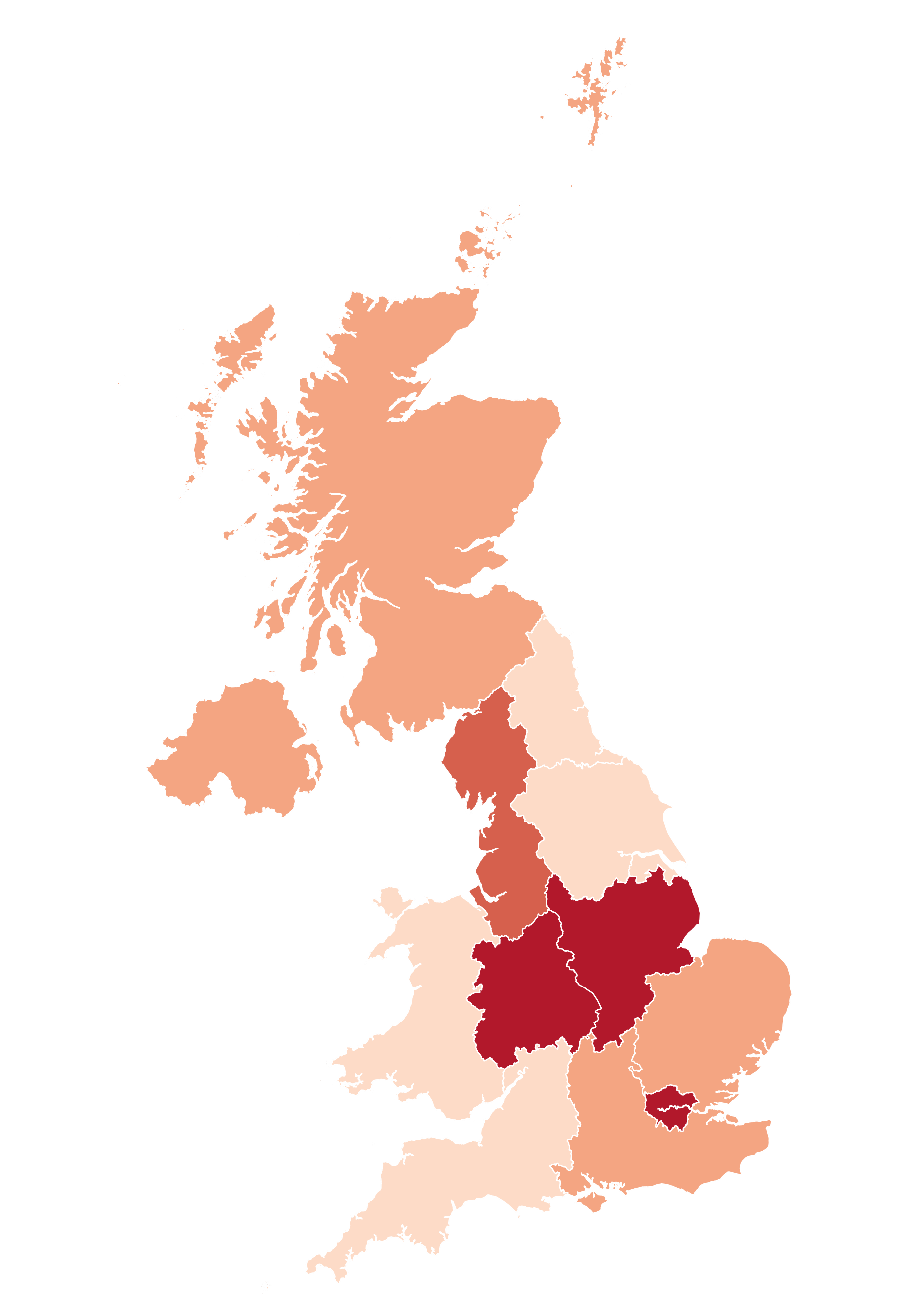


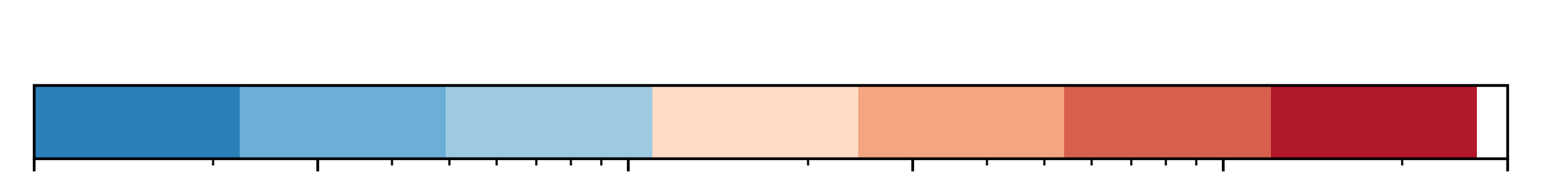


1

3

10

30

100

300

Number of Responses (Log Scale)

**Supplementary Figure 2. UK Representation of the VOICE Study.**

Choropleth map displaying the geographical distribution of VOICE respondents across UK regions, presented on a logarithmic scale (range: 1–300 responses per region).

**Supplementary Figure 3.** Breakdown of Medical Student Exposure to Assisted Dying in Education and Training

A.


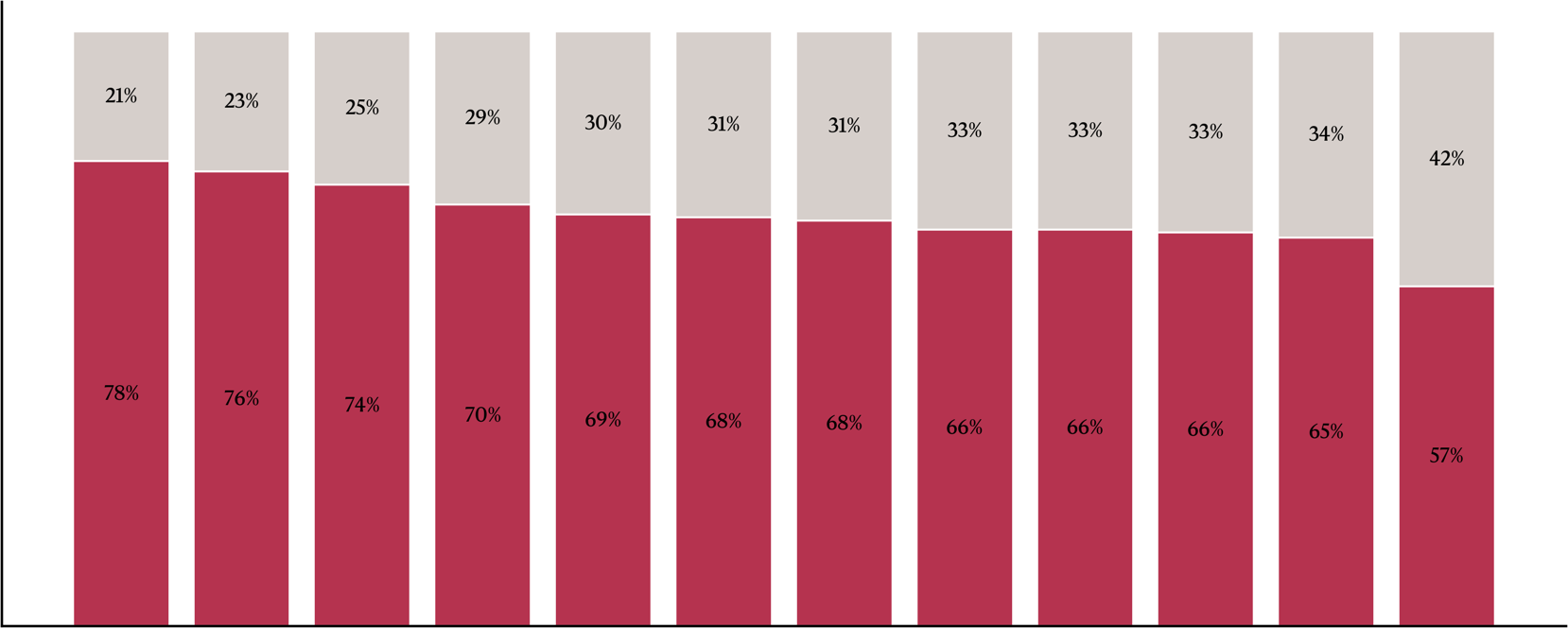

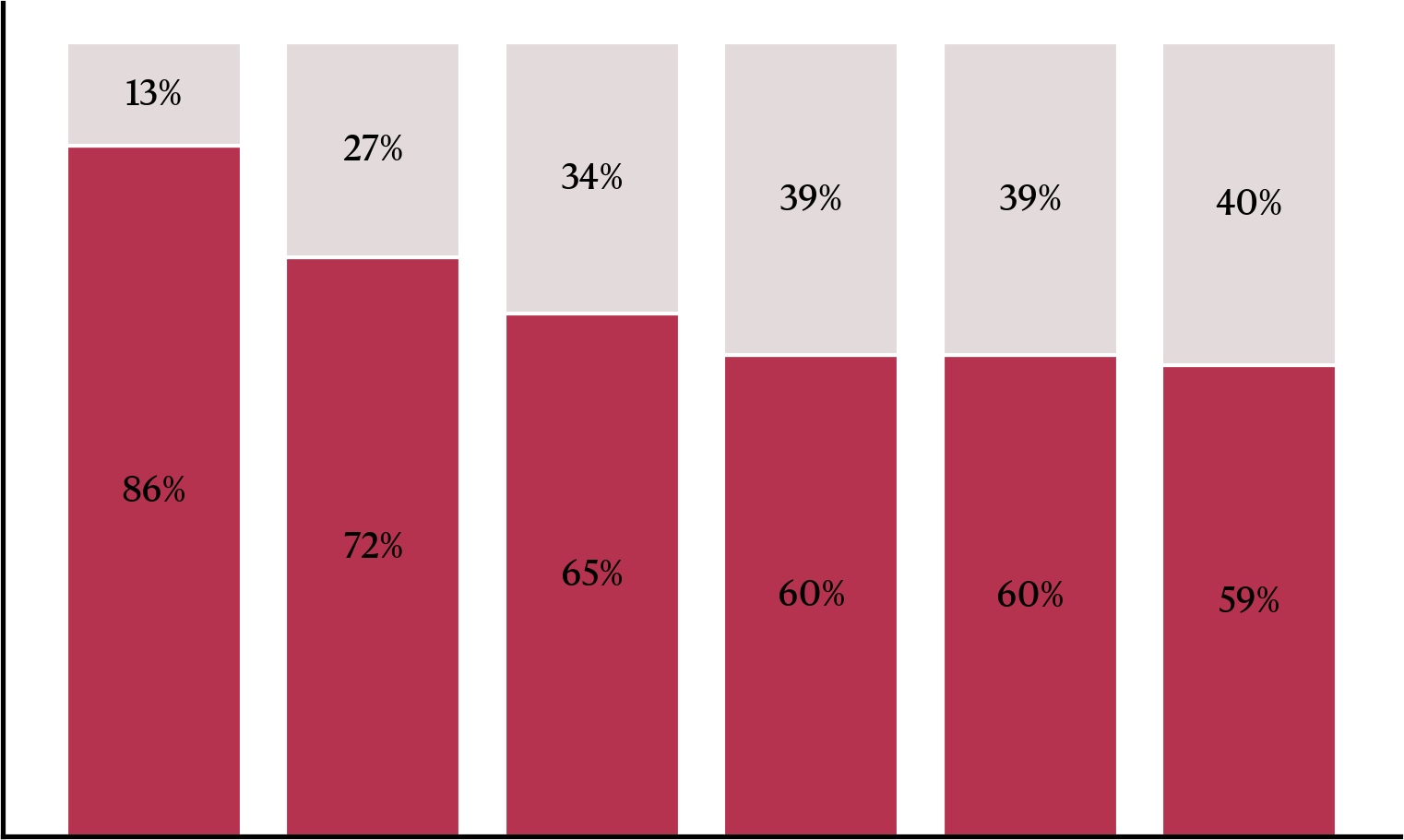


P=0.716

P<0.001

100

100

21%

23%

13%

25%

80

29%

30%

27%

42%

80

34%

39%

40%

60

60

40

78%

76%

74%

70%

40

86%

69%

72%

57%

65%

60%

60% 59%

20

20

0

0

Year 1

Year 5

Year 2

Year 6

(incl. intercalated year)

Year 3

Year 4

Year of Study

Geographical Region

Not at all or slightly covered

Moderately to extensively covered

39%

Percentage of Respondents (%)

Percentage of Respondents (%)

| 31% | 31% | 33% | 33% | 33% | 34% |
| --- | --- | --- | --- | --- | --- |
| 68% | 68% | 66% | 66% | 66% | 65% |

Q10. Based on your current experience, how confident would you feel explaining the legal, ethical, and medical implications of assisted dying to a patient or their family as a doctor?

C.


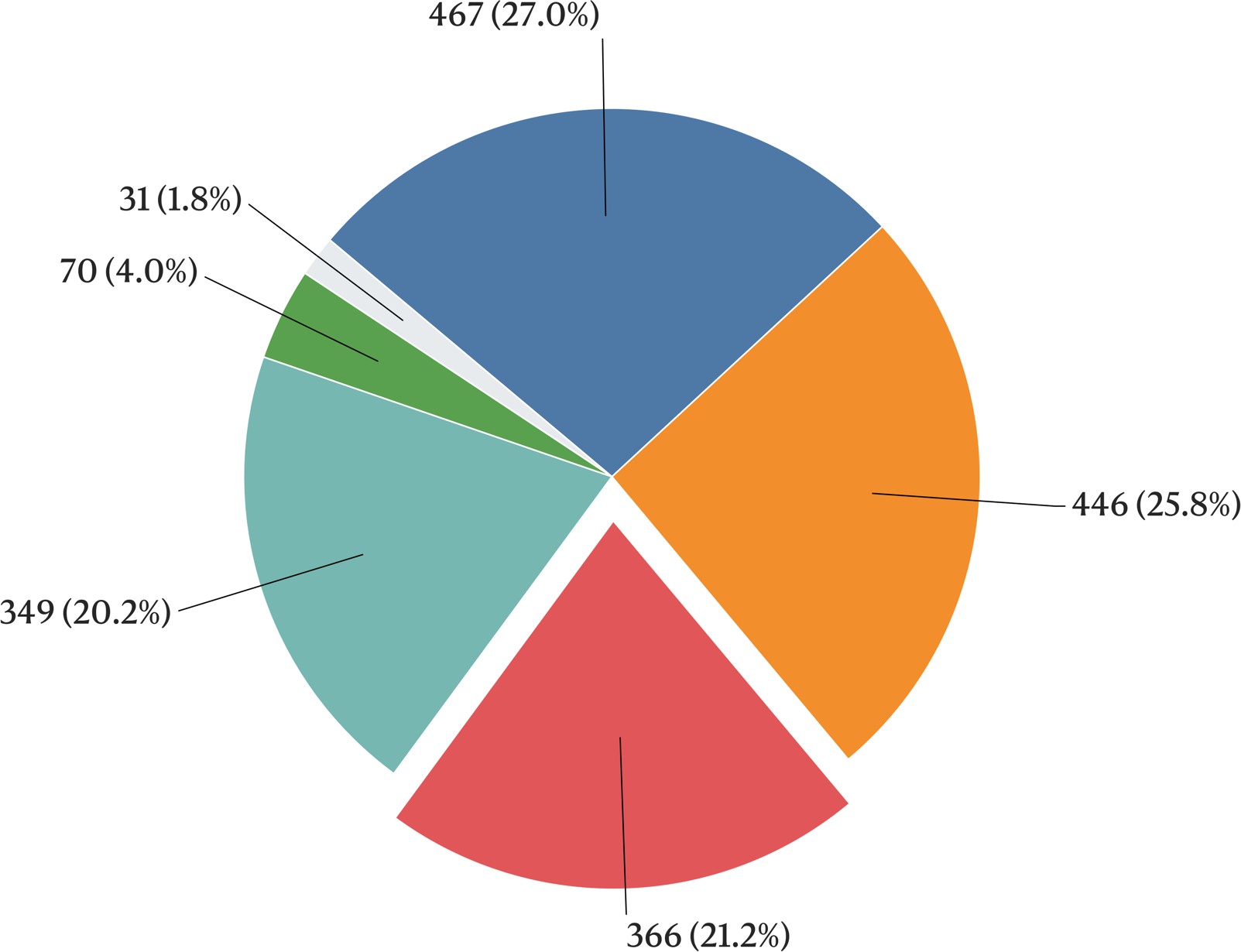


B.

467 (27.0%)

31 (1.8%)

70 (4.0%)

446 (25.8%)

349 (20.2%)

366 (21.2%)

P=0.010


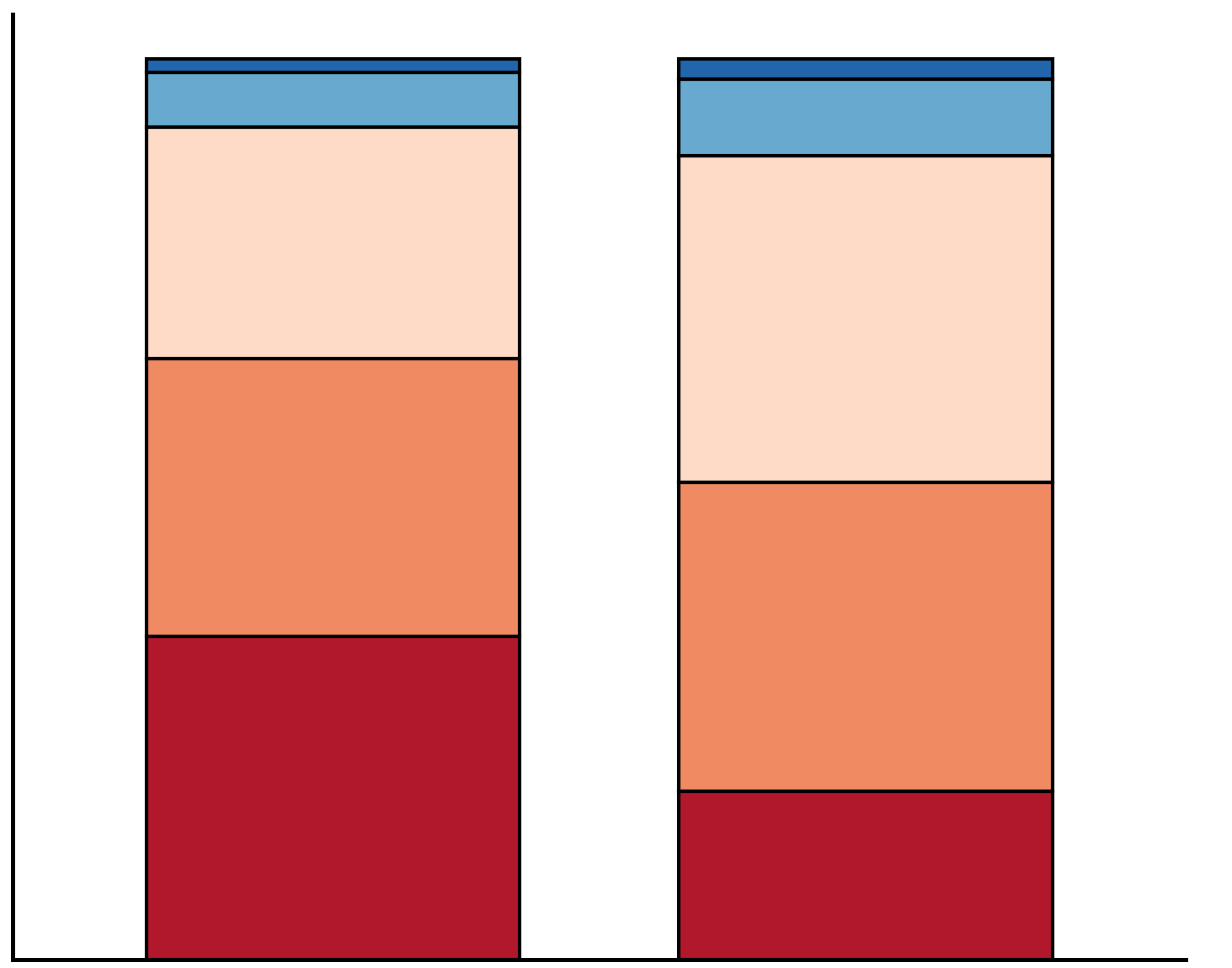
100

80

Percentage of Respondents (%)

60

40

20

Personal reading

I have not learned about assisted dying at all

0

No Yes

Taught in Medical School


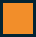
 Media coverage

North West

North East and Yorkshire

East of England

London

South East

Northern Ireland

Rep. of Ireland

South West

Wales

Other

Midlands

Scotland


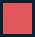
 Medical school teaching


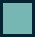
 Conversations w/ peers and mentors

Other


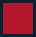
 Not at all confident
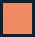
 Slightly confident

Moderately confident


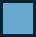
 Very confident Extremely confident

**Supplementary Figure 3. Breakdown of Medical Student Exposure to Assisted Dying in Education and Training.**

(A) Proportion of students reporting minimal (not at all or slightly covered) versus adequate (moderately to extensively covered) curricular coverage of assisted dying, by geographical region (p=0.716) and year of study (p<0.001). (B) Pie chart showing primary sources through which students report learning about assisted dying (Q8; n=1,729 total responses across 896 students; multiple responses permitted). (C) Stacked bar chart comparing self-reported clinical confidence in discussing assisted dying (Q10) between students who received formal medical school teaching and those who did not (p=0.010).

**Table 1.** Distribution of VOICE respondents by year and region of study (N = 896)

| **Group and Category** | **n** | **%** |
| --- | --- | --- |
| **Year of Study** |  |  |
| Year 1 | 219 | 24.4 |
| Year 2 | 179 | 20.0 |
| Year 3 | 189 | 21.1 |
| Year 4 | 133 | 14.8 |
| Year 5 | 87 | 9.7 |
| Year 6 (incl. intercalated) | 89 | 9.9 |
| **Region of Study** |  |  |
| East of England | 36 | 4.0 |
| London | 267 | 29.8 |
| Midlands | 176 | 19.6 |
| North East and Yorkshire | 17 | 1.9 |
| North West | 78 | 8.7 |
| Northern Ireland | 49 | 5.5 |
| Other (Please specify) | 141 | 15.7 |
| Republic of Ireland | 22 | 2.5 |
| Scotland | 28 | 3.1 |
| South East | 52 | 5.8 |
| South West | 15 | 1.7 |
| Wales | 15 | 1.7 |

**Supplementary Table 1**. Survey Instrument Used to Assess Medical Student Attitudes, Knowledge, and Ethical Reasoning on Assisted Dying

| **Q#** | **Questions** |
| --- | --- |
| 1 | Do you understand the purpose of this research project, are aware that all data collected is anonymous, and consent to have your data recorded for this study and future publications? |
| 2 | Please write full name of your referrer. |
| 3 | Which year are you in? |
| 4 | Which geographical region are you based in and where are you studying at? |
| 5 | If selected other, please specify. |
| 6 | How familiar are you with the Terminally Ill Adults (End of Life) Bill currently under consideration in the UK parliament? |
| 7 | How well do you understand the primary eligibility criteria for a patient to qualify for assisted dying under the proposed UK bill? |
| 8 | Where have you primarily learnt about assisted dying? (Select all that apply) |
| 9 | If selected other, please specify. |
| 10 | Based on your current experience, how confident would you feel explaining the legal, ethical, and medical implications of assisted dying to a patient or their family as a doctor? |
| 11 | To what extend do you feel the topic of assisted dying has been covered in your medical curriculum? |
| 12 | “Medical schools should offer specific training or modules on handling assisted dying cases.” |
| 13 | “Medical professionals should be mandated to participate in assisted dying if it becomes law.” |
| 14 | “Medical education should include ethical training on how to manage conscientious objection to assisted dying.” |
| 15 | “Assisted dying fundamentally contradicts the ethical principles of medical practice.” |
| 16 | “Assisted dying can be ethically justified in cases of terminal illness with unbearable suffering.” |
| 17 | Some critics of assisted dying express concern that vulnerable groups (e.g., elderly, disabled, mentally ill) could feel pressured to consider assisted dying. To what extend do you agree that this is a risk that needs careful regulation? |
| 18 | To what extent do you agree that there is a risk that patients in marginalised groups may feel indirect pressure to consider assisted dying if care access remains unequal? |
| 19 | If a patient requested assisted dying and met all legal criteria, how comfortable would you feel facilitating it as a doctor? |
| 20 | If legally permitted, would you personally opt out of participating in assisted dying on ethical or religious grounds? |
| 21 | Do you believe assisted dying legislation could widen or reduce existing health inequalities? |
| 22 | “Changes in assisted dying laws in other countries should inform how future developments are considered in the UK.” |
| 23 | “The UK should take into account international examples when designing safeguards for assisted dying legislation.” |
| 24 | If assisted dying were to become legal in the UK, which of the following safeguards would you support? |
| 25 | Which of the following safeguards are included in the UK’s proposed assisted dying bill? |
| 26 | Which of the following are exclusion criteria under the proposed bill? |
| 27 | Which of the following are inclusion criteria under the proposed bill? |
| 28 | Any personal thoughts, comments, ideas or concerns about assisted dying in medical education and practice – please express here. |

Supplementary Table 2. Predictors of Reporting Minimal Curriculum Coverage on Assisted Dying (Logistic Regression, n = 886)

| **Predictor** | **β (log odds)** | **aOR (95% CI)** | **p (raw)** | **p (BH-adj.)** |
| --- | --- | --- | --- | --- |
| **Year of study (reference: Year 1)** |  |  |  |  |
| Year 2 | −1.12 | 0.33 (0.18, 0.58) | <0.001 | <0.001 |
| Year 3 | −1.22 | 0.30 (0.17, 0.52) | <0.001 | <0.001 |
| Year 4 | −1.26 | 0.28 (0.16, 0.52) | <0.001 | <0.001 |
| Year 5 | −0.42 | 0.66 (0.33, 1.32) | 0.235 | 0.259 |
| Year 6 / Intercalated | −0.75 | 0.47 (0.23, 0.96) | 0.039 | 0.057 |
| **Region of training (reference: East of England)** |  |  |  |  |
| London | −0.54 | 0.58 (0.23, 1.49) | 0.260 | 0.282 |
| Midlands | −0.60 | 0.55 (0.21, 1.43) | 0.219 | 0.259 |
| North East & Yorkshire | +0.12 | 1.13 (0.25, 5.05) | 0.872 | 0.872 |
| North West | −0.57 | 0.57 (0.19, 1.65) | 0.295 | 0.309 |
| Northern Ireland | −0.25 | 0.78 (0.25, 2.41) | 0.664 | 0.696 |
| Other (specified) | −0.81 | 0.44 (0.17, 1.17) | 0.103 | 0.149 |
| Republic of Ireland | −0.51 | 0.60 (0.16, 2.34) | 0.462 | 0.490 |
| Scotland | −0.80 | 0.45 (0.13, 1.57) | 0.209 | 0.259 |
| South East | −0.88 | 0.41 (0.14, 1.26) | 0.122 | 0.168 |
| South West | −0.87 | 0.42 (0.10, 1.75) | 0.233 | 0.259 |
| Wales | −1.06 | 0.35 (0.08, 1.58) | 0.171 | 0.219 |
| **Source of learning (multi-response; each row is selected vs. not-selected, adjusted for other sources)** |  |  |  |  |
| Not learned about | −0.50 | 0.61 (0.26, 1.40) | 0.247 | 0.259 |
| Media / News | +0.01 | 1.01 (0.57, 1.77) | 0.973 | 0.973 |
| Medical School | −1.21 | 0.30 (0.19, 0.46) | <0.001 | <0.001 |
| Personal Experience | −0.10 | 0.90 (0.48, 1.72) | 0.767 | 0.779 |
| **Awareness / understanding covariates** |  |  |  |  |
| Q6: UK Bill familiarity (per 1-pt increase) | −0.46 | 0.63 (0.50, 0.79) | <0.001 | <0.001 |
| Q7: Eligibility understanding (per 1-pt) | −0.48 | 0.62 (0.49, 0.77) | <0.001 | <0.001 |
| *Model summary: Log-likelihood = −440.87; Null LL = −547.99; McFadden R² = 0.196; LLR p = 1.81 × 10⁻³³; df = 22; Hosmer–Lemeshow p > 0.20; all VIFs < 2.5.* | | | | |

**Supplementary Table 3.** Predictors of Agreement with the Ethical Justification of Assisted Dying in Terminal Illness (Logistic Regression, n = 896)

| **Predictor** | **β (log odds)** | **aOR (95% CI)** | **p (raw)** | **p (BH-adj.)** |
| --- | --- | --- | --- | --- |
| **Year of study (reference: Year 1)** |  |  |  |  |
| Year 2 | −0.30 | 0.74 (0.47, 1.16) | 0.195 | 0.367 |
| Year 3 | −0.12 | 0.89 (0.56, 1.40) | 0.615 | 0.707 |
| Year 4 | −0.42 | 0.66 (0.40, 1.07) | 0.095 | 0.261 |
| Year 5 | −0.65 | 0.52 (0.30, 0.90) | 0.022 | 0.121 |
| Year 6 / Intercalated | −0.05 | 0.95 (0.52, 1.73) | 0.872 | 0.872 |
| **Region of training (reference: East of England)** |  |  |  |  |
| London | +0.96 | 2.61 (1.26, 5.47) | 0.010 | 0.073 |
| Midlands | +1.05 | 2.86 (1.34, 6.11) | 0.007 | 0.064 |
| North East & Yorkshire | +0.70 | 2.01 (0.59, 6.75) | 0.261 | 0.409 |
| North West | +0.47 | 1.60 (0.70, 3.63) | 0.268 | 0.409 |
| Northern Ireland | +1.84 | 6.30 (2.18, 18.17) | <0.001 | 0.005 |
| Other (specified) | +0.17 | 1.19 (0.55, 2.56) | 0.663 | 0.730 |
| Republic of Ireland | −0.01 | 0.99 (0.33, 2.92) | 0.981 | 0.981 |
| Scotland | +1.96 | 7.10 (1.99, 25.28) | 0.002 | 0.022 |
| South East | +0.84 | 2.32 (0.94, 5.64) | 0.067 | 0.246 |
| South West | +0.01 | 1.01 (0.29, 3.46) | 0.990 | 0.990 |
| Wales | +1.05 | 2.86 (0.78, 10.49) | 0.113 | 0.276 |
| **Source of education (multi-response; each row is selected vs. not-selected, adjusted for other sources)** |  |  |  |  |
| Not learned about | +0.19 | 1.21 (0.66, 2.20) | 0.542 | 0.663 |
| Media / News | −0.08 | 0.92 (0.59, 1.45) | 0.718 | 0.764 |
| Medical School | +0.47 | 1.60 (1.08, 2.39) | 0.020 | 0.121 |
| Personal Experience | +0.04 | 1.04 (0.63, 1.73) | 0.870 | 0.872 |
| **Self-reported exposure / confidence** |  |  |  |  |
| Q11: Curriculum coverage (per 1-pt) | +0.03 | 1.03 (0.84, 1.25) | 0.790 | 0.827 |
| Q10: Communication confidence (per 1-pt) | +0.01 | 1.01 (0.85, 1.21) | 0.886 | 0.872 |

*Model summary: Log-likelihood = −545.49; Null LL = −574.70; McFadden R² = 0.051; LLR p = 3.83 × 10⁻⁵; df = 22; Hosmer–Lemeshow p > 0.20; all VIFs < 2.5.*

Supplementary Table 4. Predictors of Ethical or Religious Opt-Out from Assisted Dying (Logistic Regression, n = 883)

| **Predictor** | **β (log odds)** | **aOR (95% CI)** | **p (raw)** | **p (BH-adj.)** |
| --- | --- | --- | --- | --- |
| **Year of study (reference: Year 1)** |  |  |  |  |
| Year 2 | +0.22 | 1.25 (0.79, 1.97) | 0.330 | 0.500 |
| Year 3 | −0.09 | 0.91 (0.58, 1.45) | 0.700 | 0.778 |
| Year 4 | +0.26 | 1.30 (0.79, 2.14) | 0.302 | 0.488 |
| Year 5 | +0.39 | 1.48 (0.84, 2.56) | 0.171 | 0.348 |
| Year 6 / Intercalated | +0.47 | 1.60 (0.88, 2.92) | 0.122 | 0.316 |
| **Region of training (reference: East of England)** |  |  |  |  |
| London | −0.41 | 0.66 (0.32, 1.38) | 0.275 | 0.488 |
| Midlands | +0.22 | 1.25 (0.59, 2.64) | 0.565 | 0.670 |
| North East & Yorkshire | +0.49 | 1.63 (0.50, 5.31) | 0.420 | 0.551 |
| North West | +0.04 | 1.04 (0.45, 2.39) | 0.927 | 0.927 |
| Northern Ireland | −1.50 | 0.22 (0.07, 0.67) | 0.008 | 0.089 |
| Other (specified) | +0.35 | 1.42 (0.66, 3.06) | 0.369 | 0.507 |
| Republic of Ireland | −1.58 | 0.21 (0.05, 0.84) | 0.028 | 0.154 |
| Scotland | −0.45 | 0.64 (0.22, 1.88) | 0.411 | 0.551 |
| South East | −0.49 | 0.61 (0.25, 1.54) | 0.297 | 0.488 |
| South West | +0.57 | 1.77 (0.52, 6.05) | 0.365 | 0.507 |
| Wales | −1.10 | 0.33 (0.08, 1.43) | 0.138 | 0.316 |
| **Source of education (multi-response; each row is selected vs. not-selected, adjusted for other sources)** |  |  |  |  |
| Not learned about | −0.49 | 0.61 (0.33, 1.15) | 0.128 | 0.316 |
| Media / News | +0.22 | 1.25 (0.79, 1.95) | 0.352 | 0.507 |
| Medical School | +0.11 | 1.12 (0.75, 1.65) | 0.600 | 0.700 |
| Personal Experience | +0.09 | 1.09 (0.66, 1.82) | 0.740 | 0.778 |
| **Self-reported exposure / confidence** |  |  |  |  |
| Q11: Curriculum coverage (per 1-pt) | −0.16 | 0.85 (0.70, 1.03) | 0.104 | 0.316 |
| Q10: Communication confidence (per 1-pt) | −0.13 | 0.88 (0.73, 1.05) | 0.145 | 0.316 |
